# GCNFORMER: graph convolutional network and transformer for predicting lncRNA-disease associations

**DOI:** 10.1186/s12859-023-05625-1

**Published:** 2024-01-02

**Authors:** Dengju Yao, Bailin Li, Xiaojuan Zhan, Xiaorong Zhan, Liyang Yu

**Affiliations:** 1 School of Computer Science and Technology, Harbin University of Science and Technology, Harbin, 150080 China; 2 College of Computer Science and Technology, Heilongjiang Institute of Technology, Harbin, 150050 China; 3 Department of Endocrinology and Metabolism, Hospital of South, University of Science and Technology, Shenzhen, 518055 China

**Keywords:** LncRNA-disease association prediction, Graph convolutional network, transformer, Machine learning, Multiheaded attention mechanism

## Abstract

**Background:**

A growing body of researches indicate that the disrupted expression of long non-coding RNA (lncRNA) is linked to a range of human disorders. Therefore, the effective prediction of lncRNA-disease association (LDA) can not only suggest solutions to diagnose a condition but also save significant time and labor costs.

**Method:**

In this work, we proposed a novel LDA predicting algorithm based on graph convolutional network and transformer, named GCNFORMER. Firstly, we integrated the intraclass similarity and interclass connections between miRNAs, lncRNAs and diseases, and built a graph adjacency matrix. Secondly, to completely obtain the features between various nodes, we employed a graph convolutional network for feature extraction. Finally, to obtain the global dependencies between inputs and outputs, we used a transformer encoder with a multiheaded attention mechanism to forecast lncRNA-disease associations.

**Results:**

The results of fivefold cross-validation experiment on the public dataset revealed that the AUC and AUPR of GCNFORMER achieved 0.9739 and 0.9812, respectively. We compared GCNFORMER with six advanced LDA prediction models, and the results indicated its superiority over the other six models. Furthermore, GCNFORMER's effectiveness in predicting potential LDAs is underscored by case studies on breast cancer, colon cancer and lung cancer.

**Conclusions:**

The combination of graph convolutional network and transformer can effectively improve the performance of LDA prediction model and promote the in-depth development of this research filed.

## Introduction

The majority of transcribed sequences, classified as non-coding RNAs, do not possess the coding capacity for proteins. Specifically, we designate those non-coding RNAs exceeding 200 nucleotides in length as long non-coding RNAs (lncRNAs) [1–3]. For much of the past, lncRNAs were mistakenly thought of as transcription noise [4]. However, in recent times, researchers worldwide have shown a notable increase in curiosity regarding lncRNAs. Thanks to advancements in both experimental methodologies and computational prediction algorithms, the identification of thousands of lncRNAs has rapidly expanded across eukaryotic organisms, encompassing organisms from nematodes to humans. A mounting body of researches have underscored the pervasive involvement of lncRNAs throughout the cellular life cycle, operating through diverse mechanisms and exerting crucial influences on various essential biological processes [5], such as regulation of gene expression, species evolution, embryonic development, material metabolism, and tumorigenesis [6]. For example, the lncRNA MEG8 in the PANC-1 cell line in pancreatic cancer is overexpressed and represses miRNA-34a and miRNA-203 genes, leading to the upregulation of SNAIL family transcription factors and promoting the expression of calmodulin causing EMT [7–9].

In the last decade, researchers have proposed various methods for predicting potential lncRNA-disease associations (LDAs), and these approaches have demonstrated commendable performance [10]. LncRNAs and miRNAs stand out as separate classes in the domain of non-coding RNAs, each serving unique functions within the cell. Despite their divergent roles, these two RNA types exhibit intricate interconnections with one another [11]. Chen et al. delve into the advancements in addressing challenges related to the accurate prediction of miRNA-disease associations (MDAs) since 2017 [12]. Huang and Chen, along with their collaborators, conducted an extensive examination of 29 cutting-edge models designed for predicting MDAs. They propose a practical evaluation framework that can be universally applied to ensure an impartial and systematic assessment of predictive capabilities for any future models in this domain [13]. These works provide useful references for designing more effective LDA prediction models. In general, current LDA prediction methods fall under three classifications:

The first type of LDA prediction approach is based on biological networks with the premise that lncRNAs with equivalent functions are frequently connected to similar diseases [14]. LRLSLDA is the first computational model in this field, which strategically incorporates a Laplace regularization term to constrain model parameters, preventing them from becoming excessively large or small. This enhances the stability and robustness of the model, particularly in the presence of noise and data perturbations. Consequently, LRLSLDA exhibits improved performance and reliability in inferring LDAs. The introduction of LRLSLDA signifies innovative thinking and experimentation within this research field, serving as a cornerstone for subsequent developments and investigations into related models [15]. Later, Ping and colleagues devised a binary network utilizing established LDAs and inferred potential LDAs by analyzing the nature of the dichotomous network [10]. The KATZLDA model, introduced by Chen et al., first integrates Gaussian interaction profile kernel similarity, lncRNA expression similarity, lncRNA functional similarity between lncRNA and diseases, and lncRNA-disease connection networks, and then applies the KATZ algorithm to forecast lncRNAs and diseases [16]. The HGLDA establishes lncRNA-miRNA and miRNA-disease relationships and makes LDA predictions based on hypergeometric distribution tests [17]. Yu et al. combined lncRNA-miRNA, miRNA-diseases, and lncRNA-diseases associations and predicted LDAs by a double random walk model [18]. Chen et al. performed prediction by integrating the expression and semantic similarity of lncRNA and disease and using a modified version of random walk in IRWRLDA [19]. Liu et al. discovered the link by incorporating lncRNA tissue specificity and representation of both genes and lncRNAs [20]. The LNSUBRW makes predictions of potential lncRNAs with candidate diseases based on imbalanced double random walking and linear neighborhood similarity [21].

The second type of LDA prediction method employs matrix decomposition. The MFLDA is a matrix decomposition-based LDA prediction approach presented by Fu et al. [22]. This method firstly works by splitting the adjacency matrix triple factorization of combining disparate data sources into low-rank matrices, then optimizes the weight matrix by integrating heterogeneous data sources and weighting them differently to the adjacency matrix, and finally makes use of the improved low-rank. The SIMCLDA model firstly uses PCA to further extract characteristics from the similarity matrix, followed by induction matrix complementation to make predictions for LDA pairs [23]. Furthermore, Liu et al. suggested a double sparse cooperative matrix decomposition technique based on the Gaussian kernel function to forecast LDAs [24]. Xuan et al. proposed the PMFILDA which applied the probability distribution matrix for forecasting LDA pairs [25].

The third strategy is based on machine learning. Machine learning-based approaches predict LDAs by extracting features of lncRNAs and diseases, for example, the LDAP model, formulated on SVM principles, was conceptualized by Lan and collaborators [26]. To address the difficulty of learning putative representations of lncRNAs and diseases, the DMFLDA model employed cascading hidden layers [27]. To solve the difficulty of lacking negative samples, Chen and colleagues were instrumental in the development of the LRLSLDA model, a semisupervised learning method, to find the link between lncRNAs and diseases by using two classifiers without the need for negative samples. Although the LRLSLDA reduces the prerequisites for prediction, the selection of parameters for the classifiers remains to be considered [6]. Chen et al. also proposed the ILDMSF model under the premise of fusing lncRNA similarity and disease similarity [28]. In general, machine learning-based algorithms have produced promising findings in predicting LDAs. Recently, ensemble learning strategies have also produced positive results. Zhao et al. designed the ABDA model, which predicted LDAs by using an adaptive augmentation algorithm that continuously adjusts the weighting coefficients of the residual samples to make the residual samples better trained, thus achieving better results. Zhou and colleagues employed a fusion approach, integrating gradient-augmented decision trees with logistic regression, abbreviated as GBDT-LR, for the prediction of LDAs [29]. Yao and colleagues employed a random forest approach to identify and select 100 noteworthy features, subsequently utilizing these features for the prediction of LDAs [30]. Recently, deep learning has also made significant breakthroughs in this area. Xuan et al. designed multiple LDA prediction models based on convolutional neural network (CNN), such as CNNLDA [31], LDAPred [22], GCNLDA [32], and CNNDLP [33]. In addition, the VGAELDA predicted LDAs by combining variational inference with a graph self-encoder [34].

In this paper, we proposed a novel LDA prediction model based on graph convolutional network and transformer, named GCNFORMER. Firstly, based on the correlation and similarity between lncRNAs, miRNAs and diseases, we integrated intraclass similarity and interclass correlation between them to build a graph relational adjacency matrix. Secondly, to completely obtain the features between nodes, we used a graph convolutional network for feature extraction. Finally, to obtain the global dependencies between inputs and outputs, we used a transformer with the multiheaded attention mechanism to predict potential LDAs. Under fivefold cross-validation, both the AUC (area under the ROC curve) and the AUPR (area under the precision-recall curve) reveal the GCNFORMER outperforms six other LDA prediction models. Additionally, in case studies involving breast cancer, colon cancer, and lung cancer, the GCNFORMER consistently demonstrates strong performance.

## Materials and methods

### Datasets

Dataset1 is from the work of Fu et al., which includes 240 lncRNAs, 495 miRNAs, and 412 diseases [22]. In dataset1, 2697 experimentally validated LDAs were obtained from the LncRNADisease [35] and Lnc2Cancer databases [36]. Meanwhile, 13,562 MDAs were gained from the HMDD database [37], and 1002 lncRNA-miRNA interactions were got from the starBase database [38]. Dataset2 is from LDAformer [39], which contains 665 lncRNAs, 316 diseases, 295 miRNAs, 3833 LDAs, 2108 lncRNA-miRNA interactions, and 8540 MDAs. Dataset3 is from SVDNVLDA [40], which contains 861 lncRNAs, 431 diseases, 437 miRNAs, 4518 LDAs, 4189 MDAs, and 8172 lncNRA-miRNA interactions.

### Disease semantic similarity

Disease Ontology (DO) provide downloadable ontology for integrating biological data related to human diseases. The terms in DO are organized in directed acyclic graphs (DAGs) as diseases or concepts associated with diseases [41]. DAGs have been widely used in computing disease similarity. Disease ${d}_{i}$ is defined as DAG(d) = (Col(d), E(d)), where Col(d) denotes the node-set, which consists of both the current node and its ancestor nodes, and E(d) signifies the collection of edges connecting parent and child nodes. The contribution of disease d to the ontology worth of disease W, can be determined in two phases, as follows:

1 $$\left\{\begin{array}{ll}{D}_{W}(d)=1 &\quad {\text{if}} \, {\text{d}}=W \\ {{\text{D}}}_{{\text{W}}}(d)=max\left\{\Delta *{{\text{D}}}_{T}\left({{\text{d}}}^{{{\prime}}}\right)\mid {{\text{d}}}^{{{\prime}}}\in {\text{ chidren of }} d\right\} &\quad {\text{if}} \, {\text{d}}\ne W\end{array}\right.$$

2 $${\text{DV}}({\text{W}})=\sum_{{\text{d}}\in {\text{Col}}({\text{d}})} {D}_{{\text{W}}}({\text{d}})$$

where the semantic decay factor is Δ, which usually takes the value of 0.5 so that the similarity of diseases ${d}_{i}$ and ${d}_{j}$ can be calculated by the following equation:

3 $${\text{DS}}\left({{\text{d}}}_{{\text{i}}},{{\text{d}}}_{{\text{j}}}\right)=\frac{\sum_{{\text{t}}\in {\text{Col}}\left({{\text{d}}}_{{\text{i}}}\right)\cap {\text{Col}}\left({{\text{d}}}_{{\text{j}}}\right)} \left({{\text{D}}}_{{d}_{{\text{i}}}}({\text{d}})+{{\text{D}}}_{{d}_{{\text{j}}}}({\text{d}})\right)}{{\text{DV}}\left({{\text{d}}}_{{\text{i}}}\right)+{\text{DV}}\left({{\text{d}}}_{{\text{j}}}\right)}$$

### LncRNA/miRNA functional similarity

In terms of functionality, lncRNAs/miRNAs that share similarities are typically linked to comparable diseases [42]. Based on the previous work, we assume that lncRNAs or miRNAs ${z}_{1}$ and ${z}_{2}$, are associated with p and q diseases, respectively. One of them can be regarded as ${d}_{i}$ (1 ≤ i ≤ p) and ${d}_{j}$ (1 ≤ j ≤ q). As a result, the functional similarity between ${z}_{1}$ and ${z}_{2}$ can be determined using the below equation:

4 $$\begin{array}{c}Sim\left({z}_{1},{z}_{2}\right)=\frac{1}{{\text{p}}+{\text{q}}}\left[\sum_{i=1}^{{\text{p}}} \underset{1\le j\le q}{max} \left({\text{DS}}\left({{\text{d}}}_{{\text{i}}},{{\text{d}}}_{{\text{j}}}\right)\right)\left.+\sum_{j=1}^{{\text{q}}} \underset{1\le i\le p}{max} \left({\text{DS}}\left({{\text{d}}}_{{\text{j}}},{{\text{d}}}_{{\text{i}}}\right)\right)\right]\right.\end{array}$$

### Model framework

**Fig. 1:**
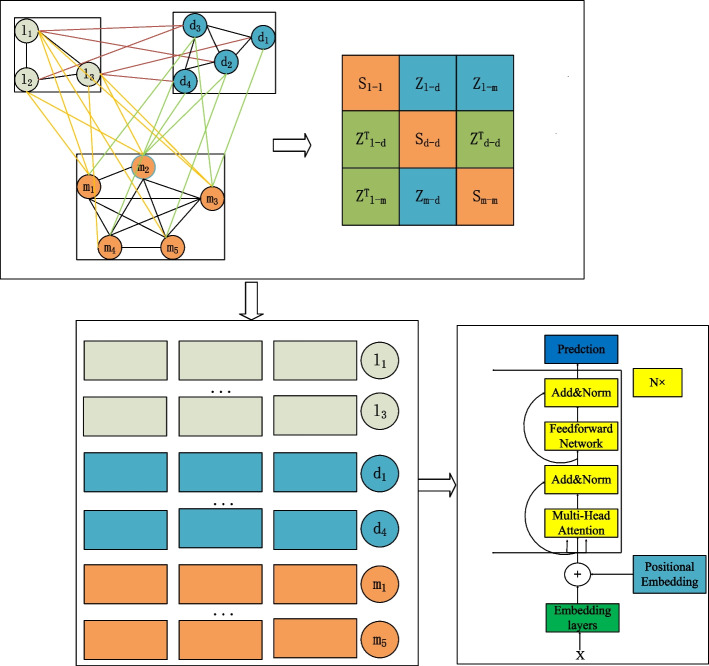
The flowchart of constructing the GCNFORMER model

This paper introduced a novel LDA prediction model, GCNFORMER, with its construction outlined in Fig. 1. Firstly, we constructed a graph relationship adjacency matrix based on the intraclass and interclass relationships between lncRNA, miRNA and disease. Secondly, according to the above graph adjacency matrix, the features between the three entities are further extracted by the GCN. Finally, we adopt the encoder part of the transformer with its own multiheaded attention mechanism to forecast associations between lncRNAs and diseases.

### Graph convolutional network

Deep learning has grown in popularity in computational biology in recent years, in which the graph convolutional network (GCN), in essence, is a feature extractor. GCN has excellent graph data processing recognition ability, and it can identify node information and the relationship between the nodes [43]. In this work, we used a GCN for feature extraction. We constructed a graph network consisting of six types of graphs, including intraclass similarity of nodes between lncRNA and lncRNA, miRNA and miRNA, disease and disease, and interclass associations between lncRNA and disease, lncRNA and miRNA, miRNA and disease. Specifically, a weighted complex graph ${{\text{B}}}_{{\text{complex}}}=(V,E)$ constructed, where V is the set of nodes consisting of lncRNA, miRNA, and disease nodes, E is the set of edges between nodes. We define ${X}_{{\text{complex}}}=(Z,S)\in {{\text{R}}}^{{{\text{N}}}_{{\text{t}}}\times {{\text{N}}}_{{\text{t}}}}$, ${{\text{N}}}_{{\text{t}}}={N}_{{\text{l}}}+{{\text{N}}}_{{\text{d}}}+{{\text{N}}}_{{\text{m}}}$, as the adjacency matrix of ${{\text{B}}}_{{\text{complex}}}$, where S is the similarity association matrix of the same nodes, while Z is the association matrix of different types of nodes.

5 $${{\text{X}}}_{{\text{complex}}}=\left[\begin{array}{ccc}{{\text{S}}}_{{\text{lncRNA}}-{\text{lncRNA}}}& {{\text{Z}}}_{{\text{lncRNA}}-{\text{disease}}}& {{\text{Z}}}_{{\text{lncRNA}}-{\text{miRNA}}}\\ {{\text{Z}}}_{{\text{lncRNA}}-{\text{disease}}}^{{\text{T}}}& {{\text{S}}}_{{\text{disease}}-{\text{disease}}}& {{\text{Z}}}_{{\text{miRNA}}-{\text{disease}}}^{{\text{T}}}\\ {{\text{Z}}}_{{\text{lncRNA}}-{\text{miRNA}}}^{{\text{T}}}& {{\text{Z}}}_{{\text{miRNA}}-{\text{disease}}}& {{\text{S}}}_{{\text{miRNA}}-{\text{miRNA}}}\end{array}\right]$$

where S denotes intraclass similarity including matrices of miRNA-miRNA similarity, disease-disease similarity, and lncRNA-lncRNA similarity; Z denotes interclass association matrix, if there is an association, it is set to 1, otherwise, it is set to 0; ${{\text{Z}}}^{{\text{T}}}$ denotes the transpose matrix of the Z matrix. After that, we set the row normalized adjacency matrix ${X}_{{\text{complex}}}$ as the feature matrix ${{\text{X}}}_{{\text{feature}}}$.

6 $${X}_{\text{feature}}=\left[\begin{array}{c}{X}^{{\text{l}}}\\ {{\text{X}}}^{\text{d}}\\ {{\text{X}}}^{\text{m}}\end{array}\right]$$

where ${{\text{X}}}_{{\text{feature}}}$ is an ${{\text{N}}}_{{\text{t}}}$×${{\text{N}}}_{{\text{t}}}$ matrix where each row is the eigenvector of a node in t. Firstly, define the following matrix as the adjacency matrix with self-connections $\widehat{{X}_{{\text{complex}}}}$, where I is the unit matrix:

7 $$\widehat{{X}_{{\text{complex}}}}={X}_{{\text{complex}}}+I$$

Then symmetric Laplace normalisation of $\widehat{{X}_{{\text{complex}}}}$ yields $\widetilde{{X}_{{\text{complex}}}}$∈${R}^{N\times N}$:

8 $$\widetilde{{X}_{{\text{complex}}}}={E}^{-\frac{1}{2}}\widehat{{X}_{{\text{complex}}}}{E}^{-\frac{1}{2}}$$

In the above equation, E∈${R}^{N\times N}$ is the diagonal matrix, and the matrix E is actually the degree matrix of $\widehat{{X}_{{\text{complex}}}}$, similar to the following equation:

9 $${E}_{{\text{ii}}}=\sum_{{\text{j}}} {\widehat{{X}_{{\text{complex}}}}}_{{\text{ij}}}$$

The matrix $\widetilde{{X}_{{\text{complex}}}}$ as well as the feature matrix ${X}_{{\text{feature}}}$ are used as inputs to the graph convolutional network, through which the network representation between lncRNA, miRNA and disease is obtained:

10 $$Z=f\left({X}_{{\text{feature}}},\widetilde{{X}_{{\text{complex}}}}\right)={\text{Softmax}}(\widetilde{{X}_{{\text{complex}}}}{X}_{{\text{feature}}}{\text{W}})$$

where W is a weight matrix, n is a hyperparameter and the operation of multiplying the matrix $\widetilde{{X}_{{\text{complex}}}}{X}_{{\text{feature}}}$ can be interpreted as the integration of spatial information. Assuming that K = $\widetilde{{X}_{{\text{complex}}}}{X}_{{\text{feature}}}$∈${R}^{{\text{N}}\times {\text{N}}}$, where ${{\text{K}}}_{i}\in {R}^{{\text{N}}}$, the *i*th row of matrix K can be understood as the feature vector of the *i*th node. By multiplying K with the weight matrix W, the node can be mapped to a low-dimensional vector ${{\text{Z}}}_{{\text{i}}}$∈${{\text{R}}}^{{\text{n}}}$, similar to Fig. 1, where the second row ${{\text{Z}}}_{2}$ as well as the third row ${{\text{Z}}}_{3}$ are representations of lncRNA ${{\text{l}}}_{2}$ as well as the disease ${{\text{d}}}_{1}$, respectively.

### Transformer

Inspired by Zhou et al., we used a transformer for the final prediction [39]. Transformer is a model that uses the attention mechanism to expedite model training. It performs well in parallelizing the computation and understanding the relationship of data. Transformer does away with conventional CNN and RNN, and the entire network is made up of attention mechanisms. Transformer adds the concept of a multiheaded attention mechanism to further improve the performance of the self-attentive layer and to address the gradient vanishing issue. The transformer also uses a residual neural network structure.

### Multihead attention

When given the same set of queries, keys, and values, multiheaded attention is a design that allows the model to learn several behaviors based on the same attention process, and then combine them. There are three inputs for the scaled dot product attention: Q, K, and V, i.e., three multiheads, which are finally spliced. Given the query ${\text{Q}}\in {{\text{R}}}^{{d}_{{\text{q}}}}$、key ${{\text{K}}\in {\text{R}}}^{{d}_{{\text{k}}}}$、value ${V\in {\text{R}}}^{{d}_{v}}$, each attention header ${{\text{X}}}_{{\text{i}}}$ (i = 1,….,X) is calculated as follows:

11 $${{\text{X}}}_{{\text{i}}}={\text{f}}\left({{\text{w}}}_{{\text{i}}}^{\left({\text{q}}\right)}{\text{q}},{{\text{w}}}_{{\text{i}}}^{\left({\text{k}}\right)}{\text{k}},{{\text{w}}}_{{\text{i}}}^{\left({\text{v}}\right)}{\text{v}}\right)\in {{\text{R}}}^{{{\text{p}}}_{{\text{v}}}}$$

The parameters that can be learned are ${{\text{w}}}_{{\text{i}}}^{\left({\text{q}}\right)}\in {{\text{R}}}^{{{\text{p}}}_{{\text{q}}}\times {{\text{d}}}_{{\text{q}}}}$, ${{\text{w}}}_{{\text{i}}}^{\left({\text{k}}\right)}\in {{\text{R}}}^{{{\text{p}}}_{{\text{k}}}\times {{\text{d}}}_{{\text{k}}}}$, ${{{\text{w}}}_{{\text{i}}}^{\left({\text{v}}\right)}\in {\text{R}}}^{{{\text{p}}}_{{\text{v}}}\times {{\text{d}}}_{{\text{v}}}}$. The output of multiheaded attention must undergo additional linear transformation to correlate to the outcome of X head splicing. The learnable parameters are ${{\text{W}}}_{0}\in {{\text{R}}}^{{{\text{p}}}_{0}\times {{\text{hp}}}_{{\text{v}}}}$:

12 $${{\text{W}}}_{0}\left[\begin{array}{c}\begin{array}{c}{{\text{x}}}_{1}\\ {{\text{x}}}_{2}\end{array}\\ {{\text{x}}}_{3}\\ .\\ .\\ .\\ {{\text{x}}}_{{\text{x}}}\end{array}\right]\in {{\text{R}}}^{{{\text{p}}}_{0}}$$

### Add and norm

The Add and Norm operations are utilized in the transformer's encoder layers, i.e., the residual join and the layer normalization operations. Residual concatenation means adding the inputs and outputs of the network, i.e.:

13 $${\text{F}}({\text{x}}) = {\text{f}}({\text{x}}) + {\text{x}}$$

When the network structure is deep, the gradient of the network backpropagation when updating the parameters, easily causes the problem of gradient disappearance, and each layer's output plus x, in the derivative of every layer adds a constant, effectively solving the problem of gradient disappearance. Compared with BatchNorm, we use LayerNorm here, which can normalize all features of each sample, and finally we can obtain:

14 $${\text{F}}({\text{x}}) = {\text{LayerNorm}}\left( {{\text{f}}({\text{x}}) + {\text{x}}} \right)$$

### Feedforward network

Although the multiheaded attention process is used to learn to articulate features, the results achieved may not be particularly good. Normalization after the attention layer, in combination with the activation function, can be better learned. The essence of feedforward neural networks is the ReLU activation function, namely:

15 $${\text{FNN}}({\text{x}})={\text{ReLU}}(0,\mathrm{ x}{w}_{1}+{{\text{b}}}_{1}){{\text{w}}}_{2}+{{\text{b}}}_{2}$$

### Prediction

The prediction score is calculated using the sigmoid activation function, and the loss function is binary cross-entropy, as shown below:

16 $${\text{Loss}}=-\Sigma [{\text{ylog}}\left({\text{p}}\right)+\left(1-{\text{y}}\right){\text{log}}\left(1-{\text{p}}\right)]$$

17 $${\text{P}}={\text{sigmoid}}({\text{WX}}+{\text{b}})$$

where p is the prediction score, sigmoid is the activation function, W denotes the weights, and b denotes the bias. If the collection contains experimental records of lncRNAs associated with disease, y = 1, otherwise y = 0.

## Experiments and results

### Fivefold cross-validation

Because of limited known LDA information and lack of unknown information, there is an imbalance problem in LDA prediction. By using cross-validation, model performance evaluation can be performed on different training and validation sets, thus minimizing the impact of imbalance. In order to objectively evaluate the performance of LDA prediction model, each fivefold cross validation experiment was performed 10 times. This is particularly important for dealing with imbalanced data because it reduces the evaluation bias caused by random sampling and ensures that the model performs consistently on multiple training and validation sets. Furthermore, the use of evaluation metrics appropriate for unbalanced data, such as AUPR, enables a thorough evaluation of the model's performance across diverse classes, thus reducing the problems associated with sample imbalance.

In fivefold cross-validation, 20% of the samples were individually taken out as test set which will not be involved in the training and validation of the model but will be used for the final evaluation of the model's performance. The remaining 80% of the samples were used as training set. In fivefold cross validation, these samples is divided into 5 equal parts. In each fold, four of these parts are used in turn to train LDA prediction model, and the remaining part is used as validation set. The performance metrics of the model were computed and recorded. This process is repeated 5 times, and the average value of fivefold is used as the final prediction result of the model. Finally, the predicted results of the trained model on the test set are used as a basis for evaluating the model's performance. Such a cross-validation approach helps in assessing the model's performance in a more comprehensive manner and reduces the effect of chance due to improper data segmentation. Additionally, to avoid over-fitting, we fine-tune the model's complexity by adjusting the number of network layers and reducing the number of units in each layer. In addition, the attention mechanism assists the model in enhancing its concentration on important parts when processing the data to prevent over-fitting.

### Evaluation indicators

By adopting fivefold cross-validation, the various evaluation indicators of LDA prediction models can be calculated. First, the receiver operating characteristic (ROC) curve can be obtained by graphing the true positive rate (TPR) and the false positive rate (FPR) at various thresholds. The TPR and FPR were determined as follows, with a tighter area under the curve near 1 indicating higher model performance:

18 $${\text{FPR}}=\frac{{\text{FP}}}{{\text{FP}}+{\text{TN}}}$$

19 $${\text{TPR}}=\frac{{\text{TP}}}{{\text{TP}}+{\text{FN}}}$$

where true positive (TP) consists of instances that are both positive and projected to be positive, false positive (FP) refers to situations that are negative but are projected to be positive, true negative (TN) refers to situations that are negative and are projected to be negative, and instances that are positive but are projected to be negative are referred to as false negatives (FN). In addition to the area the under ROC curve (AUC), the area under the PR curve (AUPR), accuracy (Acc), F1-score (F1), and Marrs correlation coefficient (Mcc) are also used to evaluate the model's performance, shown as Eqs. 20–24, where Recall represents the recall rate and Presession represents the precision rate:

20 $${\text{Precision}}=\frac{{\text{TP}}}{{\text{TP}}+{\text{FP}}}$$

21 $${\text{Recall}}=\frac{{\text{TP}}}{TP+FN}$$

22 $${\text{Acc}}=\frac{{\text{TP}}+{\text{TN}}}{{\text{TP}}+{\text{FN}}+{\text{FP}}+{\text{TN}}}$$

23 $${\text{F}}1=\frac{2\times {\text{Precision}}\times {\text{Recal}}l}{{\text{Precision}}+{\text{Recall}}}$$

24 $$Mcc=\frac{TP\times TN-FP\times FN}{\sqrt{(TP+FN)\times (TP+FP)\times (TN+FP)\times (TN+FN)}}$$

### Evaluation results

To evaluate the GCNFORMER's performance, we conducted a comparison with six contemporary methods for LDAs prediction, including IPCARF [44], GCLMTP [45], MAGCNSE [46], LR-GNN [47], VGAELDA [34], and SIMCLDA [23]. These methods include matrix decomposition-based methods, machine learning-based methods, and graph neural network-dependent methods. Specifically, GCLMTP proposes a graph comparison learning for multi-task prediction, MAGCNSE employs a multi-view graph convolutional neural network, LR-GNN is based on graph neural networks for discovering biologically significant molecular relationships, and VGAELDA integrates variational inference and graph autoencoder. In addition, IPCARF combines incremental principal component analysis and random forest algorithms. SIMCLDA is a matrix decomposition-based approach.

**Fig. 2:**
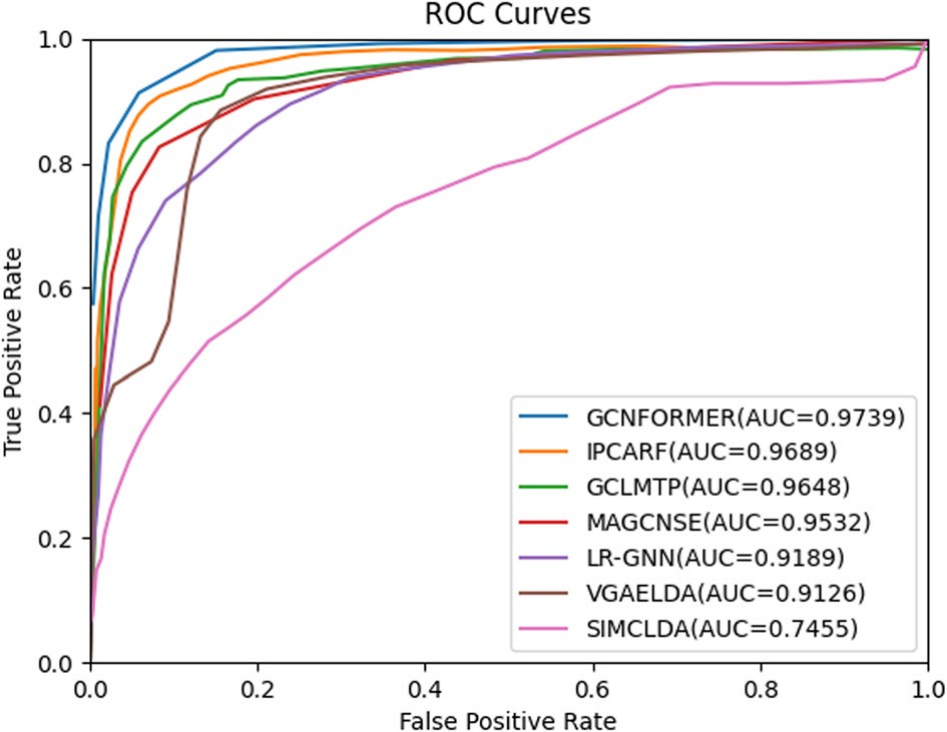
ROC curves of seven LDA prediction models on dataset 1

**Fig. 3:**
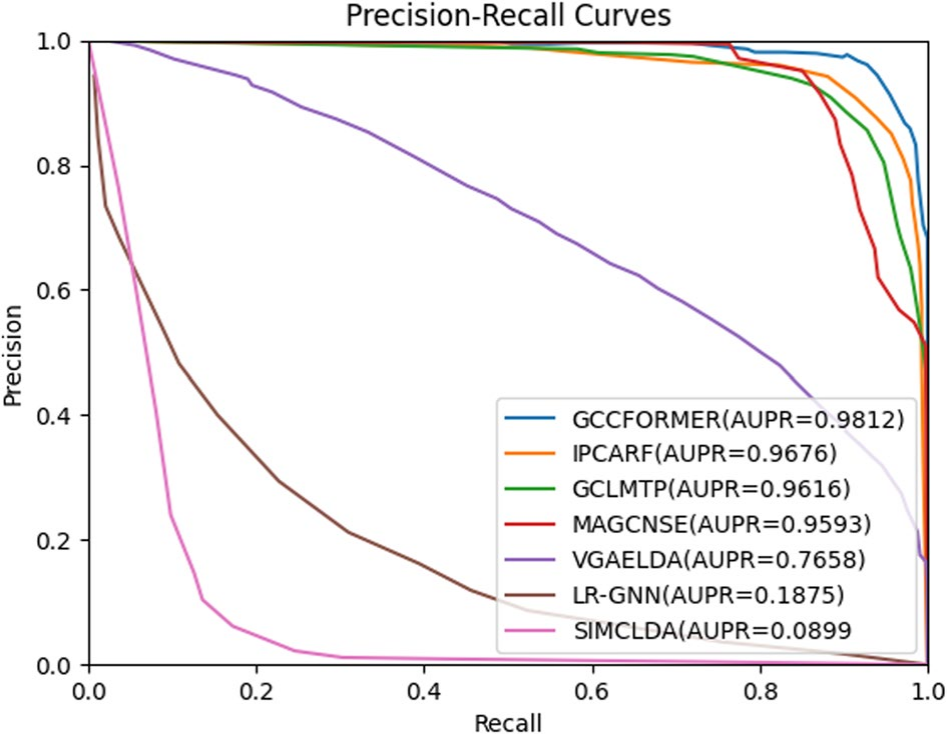
AUPR curves of seven LDA prediction models on dataset 1

In order to make a fair comparison, we determined the hyperparameters of the compared methods based on the values in the relevant literature. For IPCARF, n_estimators = 1500; for GCLMTP, the number of GCN layers was set to 2 and the node embedding dimension was set to 256; for MAGCNSE, the number of GCN layers was set to 2, the number of GCN embedding layers was set to 128, and the CNN embedding layer was set to 128; for LR-GNN, the number of GCN layers was set to 3, and embedding size is set to 64; for VGAELDA, the dimension of the output vector is 256; for SIMCLDA ${\alpha }_{l}$ is set to 0.8, ${\alpha }_{d}$ is set to 0.6, and λ is set to 1. Figures 2 and 3 display the AUC values and AUPR values obtained by all seven LDA prediction models under fivefold cross-validation, respectively. Furthermore, Table 1 lists more performance measures for the seven models involved in the comparison.

**Table 1:** The performance of seven LDA prediction models

Method	AUC	AUPR	ACC	F1	Mcc
GCNFORMER	**0.9739**	**0.9812**	**0.9726**	**0.9693**	**0.9461**
IPCARF	0.9689	0.9646	0.9093	0.9168	0.8403
GCLMTP	0.9648	0.9616	0.8992	0.9006	0.8069
MAGCNSE	0.9532	0.9593	0.9526	0.9544	0.8965
LR-GNN	0.9189	0.1875	0.8596	0.5689	0.5598
VGAELDA	0.9126	0.7658	0.9718	0.5863	0.6456
SIMCLDA	0.7455	0.0899	0.7834	0.2748	0.2376

As one can see from Figs. 2, 3 and Table 1, the average AUC and AUPR of the IPCARF model are lower than that of the GCNFORMER by 0.5% and 1.66%; the average AUC and AUPR of the GCLMTP model are 0.91% and 1.96% lower than that of the GCNFORMER; the average AUC and AUPR of the MAGCNSE are 2.07% and 2.19% lower than that of the GCNFORMER; the average AUC and AUPR of the LR-GNN are 5.5% and 79.37% lower than that of the GCNFORMER; the average AUC and AUPR of the VGAELDA model are 6.13% and 21.54% lower than that of the GCNFORMER; the average AUC and AUPR of the SIMCLDA are 22.84% and 89.13% lower than that of the GCNFORMER. These results indicate that the GCNFORMER has an excellent ability to predict LDAs.

In order to prove the generalization ability of the model, we tested the GCNFORMER on three datasets separately, and the test results are shown in Table 2, which proves that the model has good generalization ability.

**Table 2:** Performance of GCNFORMER on three datasets

Dataset	AUC	AUPR	ACC	F1	Mcc
Dataset1	0.9739	0.9812	0.9726	0.9693	0.9461
Dataset2	0.9642	0.9616	0.9196	0.9204	0.8379
Dataset3	0.9681	0.9623	0.9203	0.9289	0.8605

We also employed a two-tailed equal variance t-test to assess the performance differences between GCNFORMER and the other methods. The two-tailed equal variance t-test is a hypothesis test in statistics, which is usually used to compare whether there is a significant difference between two groups of sample means. As can be seen in Table 3, GCNFORMER outperforms the current state-of-the-art methods in both AUC and AUPR.

**Table 3:** Differences of AUC and AUPR between GCNFORMER and the other methods via t-tests

	AUC	AUPR
IPCARF	2.65847E−10	5.48979E−17
GCLMTP	5.22752E−29	1.07083E−34
MAGCNSE	1.97778E−35	1.45233E−35
LR-GNN	2.23227E−39	1.23987E−63
VGAELDA	4.54622E−43	1.94547E−53
SIMCLDA	3.3708E−54	1.53726E−64

### GCN parameter analysis

**Fig. 4:**
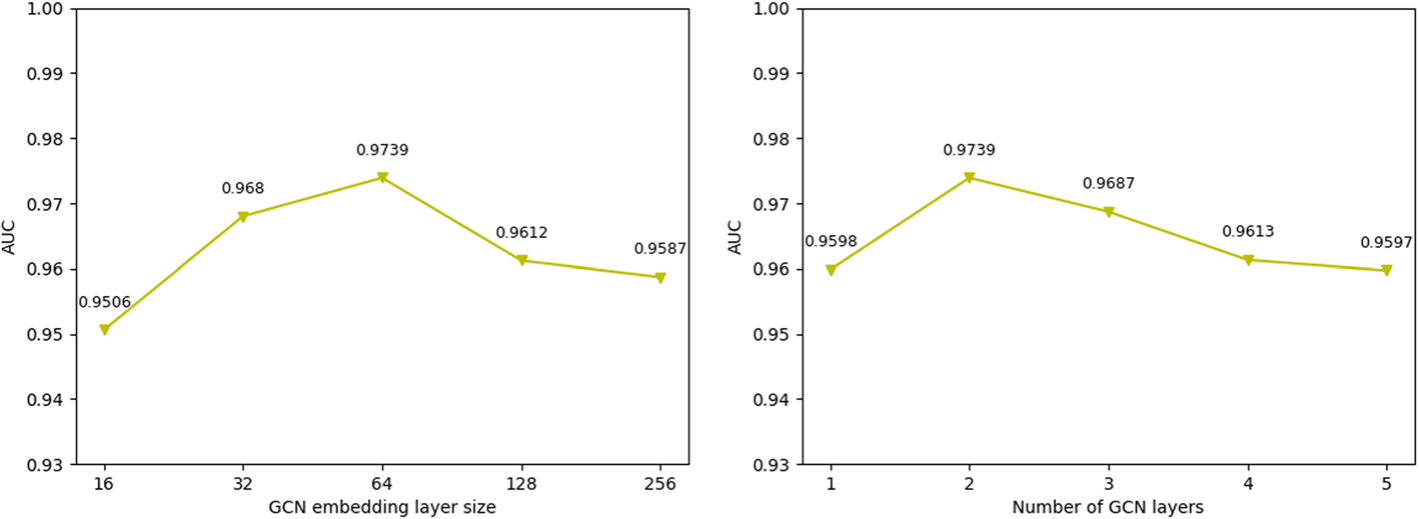
AUC results are compared for various GCN embedding sizes and layer counts

As an important module of LDA prediction, the hyperparameters of the GCN have a great influence on the prediction, and poor or too many parameter settings will affect the model's performance. Therefore, experiments are used to fine-tune the model's parameters. Figure 4 shows the evaluation of AUC values for various GCN layers and different GCN embedding sizes, which demonstrates that the model performs best when the GCN embedding size is 128 and the number of GCN layers is 2.

### Ablation study

For GCNFORMER, the interclass graph Z and the intraclass graph S contain comprehensive and detailed relationships, and we further conducted cauterization experiments to validate the importance of both interclass and intraclass similarity graphs, and eliminated modules from the transformer one by one to validate the importance of each module. Tables 4 and 5 present the outcomes of the burn-in experiments, where we observe that the best results are obtained by using both interclass association graphs and intraclass similarity graphs, and effectively validate the importance of the Add, Norm, and feed-forward network modules in the transformer. This may be because the fact that the interclass association graph better encompasses the interconnections between lncRNAs and diseases, while the intraclass similarity graph better describes the relationships between nodes, which has a crucial impact on the performance.

**Table 4:** Influence of the final result of the cauterization experiment

Interclass association Z	Intraclass similarity S	AUC	AUPR
✓	×	0.9713	0.9785
×	✓	0.9683	0.9762
✓	✓	0.9739	0.9812

**Table 5:** Influence of the final result of the cauterisation experiment

Add	Norm	FN	AUC	AUPR
×	✓	✓	0.9562	0.9654
✓	×	✓	0.9689	0.9769
✓	✓	×	0.9708	0.9776
✓	✓	✓	0.9739	0.9812

### Case studies

To conduct a more in-depth assessment of the model's effectiveness, we validated three relatively common cancers: colorectal, breast, and lung cancer using the LncRNADisease v2.0 and Lnc2Cancer v3.0 datasets and some published literature data. First, known LDAs were used as positive samples and the same negative samples were randomly selected from unknown LDAs. Next, all unknown pairs of lncRNAs associated with a specific disease were used as test samples. Finally, after training with the positive–negative samples, scores were obtained and ranked using the test samples, and evidence was sought from relevant databases.

Colon cancer stands out as one of the deadliest malignancies affecting the digestive system [48], which is a type of malignant tumor that grows in the colon, and tends to occur at the junction of the rectum and sigmoid colon. We used GCNFORMER to predict the lncRNAs linked to colon cancer, 19 of which have been supported by published research. For example, it was demonstrated that human colorectal cancer (CRC) exhibits abnormal production of long noncoding RNA cell cycle protein-dependent kinase inhibitor 2B antisense RNA1 (CDKN2B-AS1) [49]. Increased PVT1 expression is linked to colon cancer incidence, disease remission, and distant metastasis. It is also linked to increased expression of poor prognostic metastatic markers [50]. Table 6 shows the 20 lncRNAs predicted to be linked to colon cancer:

**Table 6:** Twenty predicted lncRNAs linked to colon cancer

Rank	LncRNAname	Evidence
1	CDKN2B-AS1	LncRNADiseasev2.0Lnc2Cancerv3.0
2	SNHG4	LncRNADiseasev2.0Lnc2Cancerv3.0
3	AFAP1-AS1	LncRNADiseasev2.0Lnc2Cancerv3.0
4	GAS5	LncRNADiseasev2.0Lnc2Cancerv3.0
5	HNF1A-AS1	Lnc2Cancerv3.0
6	KCNQ1OT1	Lnc2Cancerv3.0
7	BANCR	LncRNADiseasev2.0Lnc2Cancerv3.0
8	NRON	LncRNADiseasev2.0
9	TUG1	LncRNADiseasev2.0Lnc2Cancerv3.0
10	SPRY4-IT1	LncRNADiseasev2.0Lnc2Cancerv3.0
11	H19	LncRNADiseasev2.0Lnc2Cancerv3.0
12	BCYRN1	Lnc2Cancerv3.0
13	PRNCR1	LncRNADiseasev2.0Lnc2Cancerv3.0
14	CASC16	Unknown
15	PVT1	Literature
16	UCA1	LncRNADiseasev2.0Lnc2Cancerv3.0
17	XIST	LncRNADiseasev2.0Lnc2Cancerv3.0
18	TP53TG1	LncRNADiseasev2.0Lnc2Cancerv3.0
19	TUSC7	LncRNADiseasev2.0Lnc2Cancerv3.0
20	DANCR	LncRNADiseasev2.0

The most common primary malignant lung tumor is lung cancer. In the past 50 years, lung cancer incidence and mortality rates have been significantly rising worldwide, especially in industrially developed countries, and lung cancer has taken first place among male patients who died of cancer. Lung cancer, encompassing both non-small cell lung cancer (NSCLC) and small cell lung cancer (SCLC), is increasingly emerging as a leading contributor to global cancer-related mortality [51]. Table 7 summarizes the sources of evidence for lncRNAs linked to lung cancer, 18 of which have been confirmed in the literature. As an example, as determined by qPCR and protein blotting analysis, the expression level of lncRNAH19 was significantly increased in hypoxic circumstances and the invasive capacity of lung cancer was greatly increased [52]. Loss-of-function assays showed that knockdown of ZFAS1-inhibited NSCLC cell proliferation and invasive potentials increased the rate of apoptosis of NSCLC cells in vitro and attenuated tumour growth of NSCLC cells in nude mice [53].

**Table 7:** Twenty predicted lncRNAs linked to lung cancer

Rank	LncRNAname	Evidence
1	CRNDE	LncRNADiseasev2.0
2	H19	LncRNADiseasev2.0Lnc2Cancerv3.0
3	DLEU2	LncRNADiseasev2.0Lnc2Cancerv3.0
4	HOTAIR	LncRNADiseasev2.0Lnc2Cancerv3.0
5	AFAP1-AS1	LncRNADiseasev2.0Lnc2Cancerv3.0
6	NEAT1	LncRNADiseasev2.0Lnc2Cancerv3.0
7	ZFAS1	Literature
8	LINC-PINT	Lnc2Cancerv3.0
9	BCAR4	LncRNADiseasev2.0
10	TINCR	LncRNADiseasev2.0Lnc2Cancerv3.0
11	NPSR1-AS1	Lnc2Cancerv3.0
12	PANDAR	LncRNADiseasev2.0Lnc2Cancerv3.0
13	SOX2-OT	LncRNADiseasev2.0Lnc2Cancerv3.0
14	MEG3	LncRNADiseasev2.0Lnc2Cancerv3.0
15	UCA1	LncRNADiseasev2.0Lnc2Cancerv3.0
16	KIRREL3-AS3	Unknown
17	CASC16	LncRNADiseasev2.0Lnc2Cancerv3.0
18	RMST	Unknown
19	EWSAT1	LncRNADiseasev2.0Lnc2Cancerv3.0
20	CBR3-AS1	Lnc2Cancerv3.0

Despite notable strides in cancer research, breast cancer persists as a critical health concern and continues to be a prominent subject of scientific investigation. Breast cancer is the most frequent cancer in women around the world, and its prevalence and fatality rates are predicted to rise further [54]. Table 8 shows the origin of the evidence for lncRNAs linked to breast cancer, and the relevant literature has confirmed 18 of them. For example, BCAR4 expression was driven in human ZR-75–1 and MCF7 breast cancer cells, which resulted in cell proliferation [55]. Thus, a case study of colon and lung cancer and breast cancer showed that GCNFORMER has good performance in predicting relevant lncRNAs.

**Table 8:** Twenty predicted lncRNAs linked to breast cancer

Rank	LncRNAname	Evidence
1	BCAR4	LncRNADiseasev2.0Lnc2Cancerv3.0
2	XIST	LncRNADiseasev2.0Lnc2Cancerv3.0
3	UCA1	LncRNADiseasev2.0Lnc2Cancerv3.0
4	SOX2-OT	LncRNADiseasev2.0
5	HOTAIR	LncRNADiseasev2.0Lnc2Cancerv3.0
6	LINC01133	LncRNADiseasev2.0Lnc2Cancerv3.0
7	AFAP1-AS1	LncRNADiseasev2.0Lnc2Cancerv3.0
8	LINC00961	LncRNADiseasev2.0
9	MEG3	LncRNADiseasev2.0Lnc2Cancerv3.0
10	EGOT	LncRNADiseasev2.0Lnc2Cancerv3.0
11	HULC	LncRNADiseasev2.0
12	GAS5	LncRNADiseasev2.0Lnc2Cancerv3.0
13	CRNDE	Lnc2Cancerv3.0
14	CCAT2	LncRNADiseasev2.0Lnc2Cancerv3.0
15	PVT1	LncRNADiseasev2.0Lnc2Cancerv3.0
16	CDKN2B-AS1	LncRNADiseasev2.0
17	TP53COR1	Unknown
18	HOXA11-AS	LncRNADiseasev2.0Lnc2Cancerv3.0
19	MIR155HG	Unknown
20	PRNCR1	LncRNADiseasev2.0Lnc2Cancerv3.0

## Discussion

Graph convolutional network extends convolutional operations from traditional data to graph data by learning a mapping of functions through which a node can aggregate its features with those of its neighbors to achieve a more complex network, so graph convolutional network has a superior ability to process graph data. Today, the attention mechanism is frequently used in a variety of tasks, and its advantage is its ability to amplify the impact of important parts of the data. Transformer itself is a model that uses the attention mechanism to improve its effectiveness, and its multiheaded attention mechanism further refines the attention layer by enhancing the model's capacity to focus on various locations as well as giving multiple representation subspaces to increase model performance. In GCNFORMER model, graph convolutional network can effectively capture the topology and interactions in lncRNA-disease association network, while transformer can extract the contextual information under the complex relationships. Therefore, combining graph convolutional network with transformer can help to learn richer and more efficient feature representations, improve the ability to identify and mine key features in lncRNA-disease associations, and provide more accurate prediction. Taken together, the combination of graph convolutional network and transformer brings new ideas and technical means to lncRNA-disease association prediction field, improves the accuracy and explanatory ability of lncRNA-disease association prediction model, and can promote the in-depth development of the related research.

## Conclusion

In this work, we proposed a graph convolutional network and transformer-based LDA prediction method (GCNFORMER). First, we constructed graph relational adjacency matrices by combining intraclass similarity and interclass associations between lncRNAs, diseases, and miRNAs. Second, we employed a graph convolutional network to fully extract the characteristics among the nodes. Finally, we implemented a transformer encoder to forecast potential lncRNA-disease associations. The AUC, AUPR, and some other evaluation indicators under fivefold cross validation outperform six other state-of-art lncRNA-disease association prediction models. The case study on three cancers demonstrate that GCNFORMER is a useful LDA prediction model with good prediction performance. Of course, there are still some aspects which can be further improved. First, only lncRNA, miRNA, and disease information were used in current GCNFORMER model. To improve the model's effectiveness in predicting LDAs, our next step will introduce more biological information into GCNFORMER. Specifically, large-scale multi-omics data, including genomics, transcriptomics, proteomics, and clinical data, can be further integrated, and comprehensive data analyses can be carried out to identify potential LDAs. Second, in addition to LDA prediction, further in-depth studies on the function and mechanism of lncRNAs can be carried out in the future to explore their specific roles in the process of disease development, thus revealing their importance in the mechanism of disease occurrence.

## Data Availability

The data and materials are available from https://github.com/ydkvictory/GCNFORMER.

## References

[1] Djebali S, Davis CA, Merkel A, Dobin A, Lassmann T, Mortazavi A, Tanzer A, Lagarde J, Lin W, Schlesinger F, Xue C, Marinov GK, Khatun J, Williams BA, Zaleski C, Rozowsky J, Röder M, Kokocinski F, Abdelhamid RF, Alioto T, Gingeras TR (2012). Landscape of transcription in human cells. Nature.

[2] Pennisi E (2010). Shining a light on the genome's 'dark matter'. Science.

[3] Zhou S, Ding F, Gu X (2016). Non-coding RNAs as emerging regulators of neural injury responses and regeneration. Neurosci Bull.

[4] Sun W, Shi Y, Wang Z, Zhang J, Cai H, Zhang J, Huang D (2018). Interaction of long-chain non-coding RNAs and important signaling pathways on human cancers (Review). Int J Oncol.

[5] Chen X, Yan CC, Zhang X, You ZH (2017). Long non-coding RNAs and complex diseases: from experimental results to computational models. Brief Bioinform.

[6] Chen X, Yan CC, Luo C, Ji W, Zhang Y, Dai Q (2015). Constructing lncRNA functional similarity network based on lncRNA-disease associations and disease semantic similarity. Sci Rep.

[7] Mohanty V, Gökmen-Polar Y, Badve S, Janga SC (2015). Role of lncRNAs in health and disease-size and shape matter. Brief Funct Genomics.

[8] Mercer TR, Mattick JS (2013). Structure and function of long noncoding RNAs in epigenetic regulation. Nat Struct Mol Biol.

[9] Esteller M (2011). Non-coding RNAs in human disease. Nat Rev Genet.

[10] Ping P, Wang L, Kuang L, Ye S, Iqbal MFB, Pei T (2019). A Novel Method for LncRNA-disease association prediction based on an lncRNA-disease association network. IEEE/ACM Trans Comput Biol Bioinf.

[11] Chen X, Xie D, Zhao Q, You ZH (2019). MicroRNAs and complex diseases: from experimental results to computational models. Brief Bioinform.

[12] Huang L, Zhang L, Chen X (2022). Updated review of advances in microRNAs and complex diseases: taxonomy, trends and challenges of computational models. Brief Bioinform.

[13] Huang L, Zhang L, Chen X (2022). Updated review of advances in microRNAs and complex diseases: towards systematic evaluation of computational models. Brief Bioinform.

[14] Clark MB, Johnston RL, Inostroza-Ponta M, Fox AH, Fortini E, Moscato P, Dinger ME, Mattick JS (2012). Genome-wide analysis of long noncoding RNA stability. Genome Res.

[15] Chen X, Yan GY (2013). Novel human lncRNA-disease association inference based on lncRNA expression profiles. Bioinformatics.

[16] Chen X (2015). KATZLDA: KATZ measure for the lncRNA-disease association prediction. Sci Rep.

[17] Chen X (2015). Predicting lncRNA-disease associations and constructing lncRNA functional similarity network based on the information of miRNA. Sci Rep.

[18] Yu G, Fu G, Lu C, Ren Y, Wang J (2017). BRWLDA: bi-random walks for predicting lncRNA-disease associations. Oncotarget.

[19] Chen X, You ZH, Yan GY, Gong DW (2016). IRWRLDA: improved random walk with restart for lncRNA-disease association prediction. Oncotarget.

[20] Li M, Zhao B, Yin R, Lu C, Guo F, Zeng M (2023). GraphLncLoc: long non-coding RNA subcellular localization prediction using graph convolutional networks based on sequence to graph transformation. Brief Bioinform.

[21] Xie G, Jiang J, Sun Y (2022). LDA-LNSUBRW: lncRNA-Disease association prediction based on linear neighborhood similarity and unbalanced bi-random walk. IEEE/ACM Trans Comput Biol Bioinf.

[22] Fu G, Wang J, Domeniconi C, Yu G (2018). Matrix factorization-based data fusion for the prediction of lncRNA-disease associations. Bioinformatics.

[23] Lu C, Yang M, Luo F, Wu FX, Li M, Pan Y, Li Y, Wang J (2018). Prediction of lncRNA-disease associations based on inductive matrix completion. Bioinformatics.

[24] Liu JX, Gao MM, Cui Z, Gao YL, Li F (2021). DSCMF: prediction of LncRNA-disease associations based on dual sparse collaborative matrix factorization. BMC Bioinform.

[25] Xuan Z, Li J, Yu J, Feng X, Zhao B, Wang L (2019). A probabilistic matrix factorization method for identifying lncRNA-disease associations. Genes.

[26] Lan W, Li M, Zhao K, Liu J, Wu FX, Pan Y, Wang J (2017). LDAP: a web server for lncRNA-disease association prediction. Bioinformatics.

[27] Zeng M, Lu C, Fei Z, Wu FX, Li Y, Wang J, Li M (2021). DMFLDA: a deep learning framework for predicting lncRNA-disease associations. IEEE/ACM Trans Comput Biol Bioinf.

[28] Chen Q, Lai D, Lan W, Wu X, Chen B, Liu J, Chen YP, Wang J (2021). ILDMSF: inferring associations between long non-coding RNA and disease based on multi-similarity fusion. IEEE/ACM Trans Comput Biol Bioinf.

[29] Zhou S, Wang S, Wu Q, Azim R, Li W (2020). Predicting potential miRNA-disease associations by combining gradient boosting decision tree with logistic regression. Comput Biol Chem.

[30] Yao D, Zhan X, Zhan X, Kwoh CK, Li P, Wang J (2020). A random forest based computational model for predicting novel lncRNA-disease associations. BMC Bioinform.

[31] Xuan P, Cao Y, Zhang T, Kong R, Zhang Z (2019). Dual Convolutional neuralnetworks with attention mechanisms based method for predicting disease-related lncRNA genes. Front Genet.

[32] Xuan P, Pan S, Zhang T, Liu Y, Sun H (2019). Graph convolutional network and convolutional neural network based method for predicting lncRNA-disease associations. Cells.

[33] Xuan P, Sheng N, Zhang T, Liu Y, Guo Y (2019). CNNDLP: a method based on convolutional autoencoder and convolutional neural network with adjacent edge attention for predicting lncRNA-disease associations. Int J Mol Sci.

[34] Shi Z, Zhang H, Jin C, Quan X, Yin Y (2021). A representation learning model based on variational inference and graph autoencoder for predicting lncRNA-disease associations. BMC Bioinform.

[35] Chen G, Wang Z, Wang D, Qiu C, Liu M, Chen X, Zhang Q, Yan G, Cui Q (2013). LncRNADisease: a database for long-non-coding RNA-associated diseases. Nucleic Acids Res.

[36] Ning S, Zhang J, Wang P, Zhi H, Wang J, Liu Y, Gao Y, Guo M, Yue M, Wang L, Li X (2016). Lnc2Cancer: a manually curated database of experimentally supported lncRNAs associated with various human cancers. Nucleic Acids Res.

[37] Li Y, Qiu C, Tu J, Geng B, Yang J, Jiang T, Cui Q (2014). HMDD v2.0: a database for experimentally supported human microRNA and disease associations. Nucleic Acids Res.

[38] Yang JH, Li JH, Shao P, Zhou H, Chen YQ, Qu LH (2011). starBase: a database for exploring microRNA-mRNA interaction maps from Argonaute CLIP-Seq and Degradome-Seq data. Nucleic Acids Res.

[39] Zhou Y, Wang X, Yao L, Zhu M (2022). LDAformer: predicting lncRNA-disease associations based on topological feature extraction and Transformer encoder. Brief Bioinform.

[40] Li J, Li J, Kong M, Wang D, Fu K, Shi J (2021). SVDNVLDA: predicting lncRNA-disease associations by Singular Value Decomposition and node2vec. BMC Bioinform.

[41] Li J, Gong B, Chen X, Liu T, Wu C, Zhang F, Li C, Li X, Rao S, Li X (2011). DOSim: an R package for similarity between diseases based on Disease Ontology. BMC Bioinform.

[42] Yang Q, Li X (2021). BiGAN: LncRNA-disease association prediction based on bidirectional generative adversarial network. BMC Bioinform.

[43] Barr WA, Sheth RB, Kwon J, Cho J, Glickman JW, Hart F, Chatterji OK, Scopino K, Voelkel-Meiman K, Krizanc D, Thayer KM, Weir MP (2020). GCN sensitive protein translation in yeast. PLoS ONE.

[44] Zhu R, Wang Y, Liu JX, Dai LY (2021). IPCARF: improving lncRNA-disease association prediction using incremental principal component analysis feature selection and a random forest classifier. BMC Bioinform.

[45] Sheng N, Wang Y, Huang L, Gao L, Cao Y, Xie X, Fu Y (2023). Multi-task prediction-based graph contrastive learning for inferring the relationship among lncRNAs, miRNAs and diseases. Brief Bioinform.

[46] Liang Y, Zhang ZQ, Liu NN, Wu YN, Gu CL, Wang YL (2022). MAGCNSE: predicting lncRNA-disease associations using multi-view attention graph convolutional network and stacking ensemble model. BMC Bioinform.

[47] Kang C, Zhang H, Liu Z, Huang S, Yin Y (2022). LR-GNN: a graph neural network based on link representation for predicting molecular associations. Brief Bioinform.

[48] Bray F, Ferlay J, Soerjomataram I, Siegel RL, Torre LA, Jemal A (2018). Global cancer statistics 2018: GLOBOCAN estimates of incidence and mortality worldwide for 36 cancers in 185 countries. CA Cancer J Clin.

[49] Pan J, Lin M, Xu Z, Xu M, Zhang J, Weng Z, Lin B, Lin X (2021). CDKN2B antisense RNA 1 suppresses tumor growth in human colorectal cancer by targeting MAPK inactivator dual-specificity phosphatase 1. Carcinogenesis.

[50] Luo Z, Chen R, Hu S, Huang X, Huang Z (2022). PVT1 promotes resistance to 5-FU in colon cancer via the miR-486-5p/CDK4 axis. Oncol Lett.

[51] Wu F, Wang L, Zhou C (2021). Lung cancer in China: current and prospect. Curr Opin Oncol.

[52] Li H, Wang J, Jin Y, Lin J, Gong L, Xu Y (2022). Hypoxia upregulates the expression of lncRNA H19 in non-small cell lung cancer cells and induces drug resistance. Transl Cancer Res.

[53] Fernandez-Cuesta L, Thomas RK (2015). Molecular pathways: targeting NRG1 fusions in lung cancer. Clin Cancer Res.

[54] Anastasiadi Z, Lianos GD, Ignatiadou E, Harissis HV, Mitsis M (2017). Breast cancer in young women: an overview. Updat Surg.

[55] Godinho M, Meijer D, Setyono-Han B, Dorssers LC, van Agthoven T (2011). Characterization of BCAR4, a novel oncogene causing endocrine resistance in human breast cancer cells. J Cell Physiol.

